# Increasing Access to Care for the Underserved: Voices of Riders, Drivers, & Staff of a Rural Transportation Program

**DOI:** 10.3390/ijerph192013539

**Published:** 2022-10-19

**Authors:** Abby J. Schwartz, Alice R. Richman, Mallary Scott, Haiyong Liu, Weyling White, Caroline Doherty

**Affiliations:** 1 School of Social Work, East Carolina University, Greenville, NC 27858, USA; 2 Department of Health Education and Promotion, East Carolina University, Greenville, NC 27858, USA; 3 Department of Finance and Economics, Texas State University, San Marcos, TX 78666, USA; 4 Care Share Health Alliance, Raleigh, NC 27609, USA; 5 Roanoke Chowan Community Health Center, Ahoskie, NC 27910, USA

**Keywords:** transportation, healthcare access, health disparities, social determinants of health

## Abstract

The qualitative data presented in this paper was part of a larger concurrent mixed methods study evaluating the effectiveness of a transportation program (Project TRIP) for low-income residents in rural eastern North Carolina. Twenty stakeholders involved in TRIP were interviewed, including riders (n = 12) of which 83% were over 50 years old, program staff including the program coordinator and 5 case managers (n = 6), and transportation providers (n = 2). Due to the COVID-19 pandemic, interviews were completed by phone with each participant. Themes from the qualitative data included the: (1) Emotional, health, & financial impacts of TRIP, (2) Changes that should be implemented into TRIP when replicating the program, and (3) Unique aspects of how TRIP operates that could inform other rural transportation programs. Thematic analysis was used to analyze the transcript data. The findings are couched in the context of how TRIP potentially defrays the impacts of cumulative disadvantage that residents experience over the life course by increasing access to healthcare.

## 1. Introduction

**Figure 1:**
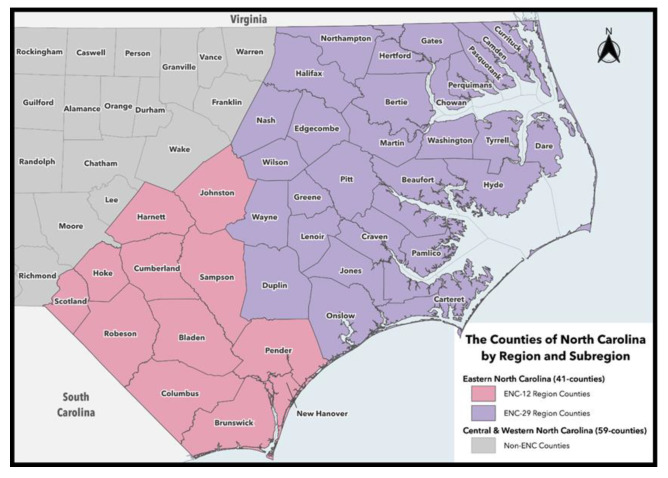
Eastern NC eNC-29 region counties. Map provided by the ECU Center for Geographic Information Science (https://gis.ecu.edu).

In North Carolina (NC), approximately 14.0% of the population was at 100% Federal Poverty Level (FPL) in 2019, and 6.5% of households did not have access to a vehicle in 2017 (based on a count of vehicles at home that is available to be used) [1,2]. The FPL Guidelines for 2019 provided by the United States (U.S.) Health and Human Services (HHS) are based on household size and income. For example, a household of 4 people in 2019, with an annual income of $25,750 would be considered at 100% FPL [3]. The percentages of FPL and lack of access to a vehicle increase in the eastern part of the state, particularly the 29 county sub-region (eNC-29), the current study setting (Figure 1). Compared to the rest of NC, 25 of the counties in eNC-29 are classified as rural [4], 24 counties have greater than 13.6% of residents at 100% FPL, and 20 counties have greater than 6.5% of households with no access to vehicles [1].

In eNC-29 17.1% of residents are below the poverty level, with disparities most prominent among residents who are Black, Indigenous, and People of Color (BIPOC) [4]. Of the counties that comprise eNC-29, as of 2022, 62.1% (n = 18) are classified as Tier 1 areas, indicating the “most distressed”, based on unemployment rates, median household income, population growth, and adjusted property tax base per capita [5].

Counties in the eNC-29 subregion are aging, akin to other studies examining rural populations. According to 2020 United States Census data, the median age of residents in eNC-29 was 43.4, higher than the median age across the state of North Carolina (38.9) [4]. 

Rural residents are older, require vehicles that support mobility needs, and have been identified as not as likely to travel due to health conditions compared to urban counterparts [6]. Health disparities among rural adults have been identified such as health inequities varying by race/ethnicity [7,8], and increased risk of dementia and cognitive impairment among older rural residents [9]. Jensen et al. (2020) have called for greater attention to “how contextual conditions (e.g., pollution, crime, security, walkability, aesthetics) affect mental and cognitive health among rural populations…[and] within-rural disparities in health and aging” (p. 1329) [10]. Transportation, one social determinant of health and contextual factor, has been identified as supporting or impeding access to places that support health [11,12,13,14]. Additional barriers such as healthcare costs, being uninsured/underinsured, navigating a fragmented healthcare system, and an inability to afford transportation are associated with negative health consequences (e.g., missed appointments, not filling prescriptions) [11,13,15]. 

For additional context, in the U.S. individuals must be eligible to receive free transportation such as through Medicaid with eligibility often tied to having a lower income level [16]. However, there remain individuals who are underinsured or uninsured, and do not have the financial means and/or access to transportation [15] such as a personal vehicle, or other transportation services such as bus, subway, light rail fare or other transportation options. A study completed by the U.S. Government Accountability Office found that coordination of non-emergency medical transportation (NEMT) programs in the United States is fragmented, with the majority of spending related to transportation for Medicaid recipients (individuals identified as impoverished) [16]. Non-emergency medical transportation typically transports riders to their scheduled destination and brings the individual back home. However, this could include extended wait times for pick up and drop off to and from appointments [17]. In rural areas the need for expanded services has been identified, yet transportation may not be available at all or may be limited in the distances it will travel [11,18]. Some patients hesitate asking family or friends for a ride to not burden them, which in some cases could mean the person needing to take off work to transport the patient to an appointment [11,17]. In addition to gas, lodging, etc., costs of trips to other areas for care can be costly. Due to these challenges, some patients may decide to forgo attending appointments altogether [11,19].

Interventions to address the need for transportation have been completed, as described in 2 systematic reviews [20,21]. Starbird et al. (2019) examined interventions that included an element of chronic care transportation. The articles reviewed were from the U.S., Canada, and Uganda. The authors found that transportation supports (e.g., bus tickets, free shuttle), were associated with improved outcomes (e.g., attending follow-up appointments) [21]. Solomon et al.’s (2020) systematic review examined U.S. NEMT interventions aimed at impacting healthcare utilization and outcomes. The authors found varied results. Some studies showed an association between transportation health care utilization/health outcomes, while others did not. The authors noted that this could have been attributed to not targeting patients who had an identified need for transportation [20]. Thus, transportation to improve access to health care, healthcare utilization and health outcomes, is an area where more research is needed with particular focus on specific vulnerable populations in need of transportation. 

Some of the interventions examined in the systematic reviews above seem promising in intervening in the health trajectory of individuals’ lives, potentially placing them on a path towards improved health outcomes. Cumulative disadvantage theory “stresses the way in which personal or social trajectories are formed from the interplay of biological, behavioral, and social-structural forces” (Ferraro, 2001, p. 324) [22]. This theory is particularly important in informing the present research and is particularly relevant given the study setting of the rural southeastern U.S. and the poverty experienced by rural eNC residents. 

### 1.1. Key Components of TRIP

Project TRIP (Transporting Residents with Innovative Practices) is one response to the barriers rural eNC-29 residents face in accessing health care. Funded by the Roanoke Chowan Foundation, TRIP began in 2015 and provides free individualized transportation to health care appointments, pharmacies, grocery stores/food banks, and other places that support health and well-being. TRIP includes four key components: (1) Individual rides (no ride sharing), (2) No wait times, as drivers wait for the patient until their appointment is complete and then bring them directly home, (3) Rides to geographical areas that public transportation may not travel to, thus providing patients access to a greater number of healthcare providers that may better meet their needs, and (4) Locations patients are transported to include medical appointments, pharmacies, grocery stores, and other health-related places [23]. As of March 2022, TRIP transported patients in Bertie, Hertford, Northampton, and Gates counties, with program coordination taking place at Roanoke Chowan Community Health Center (RCCHC). Patient eligibility includes being at 200% FPL. An example of a household of 4 at 200% FPL means that the household annual income is $55,500 (in 2022). Although higher than 100% FPL, that household would remain at poverty level as defined by HHS [24]. Another criterion for eligibility is that there is a need for transportation as identified through the social determinants of health assessment during patient intake and assessment. At enrollment patients sign a responsibilities form that outlines aspects of the program that they understand and agree to. For example, patients understand and agree that they will be ready and on time for each scheduled pick up or provide at least 24 hours’ notice to cancel their pickup.

### 1.2. Goal of the Present Study

RCCHC’s initial Executive Summary with outcomes regarding TRIP indicated potential program effectiveness with decreased healthcare utilization among patients [23], however, a formal evaluation had not been completed to assist in sustaining and scaling TRIP to other counties in the region. The present study included a mixed methods concurrent evaluation of TRIP with the goal of determining TRIP’s effectiveness, efficiency, impact, and sustainability. To meet this goal, one aspect included qualitative interviews with TRIP stakeholders including staff/case managers, patients/riders, and transportation companies. The present paper provides the themes that emerged from the stakeholder qualitative interviews and contributes to answering the question of how a lack of access to healthcare-related services in rural communities for can be circumvented by a program like TRIP.

## 2. Materials and Methods

### 2.1. Settings and Participants

Twenty stakeholders associated with Project TRIP were interviewed via telephone due to the COVID-19 pandemic. Participants included 6 staff members (5 case managers and 1 project coordinator), 2 transportation company owners who also served as drivers, and 12 patients who received rides through TRIP. Staff and transportation company individuals were identified by RCCHC community partners collaborating on this project (purposively sampled). Multiple approaches were used to sample riders to interview including purposive and random sampling. A consent to contact script and form was used by RCCHC staff to ask patients/riders they had recent communications with if they could be contacted by the researchers for an interview. The authors also had a list of TRIP riders, and using a random number generator identified riders to call to consent to contact. The third author (MS) was responsible for this task and informed the first and second author of any riders who agreed to be contacted. If a patient/rider consented, they were then contacted by a person on the research team to be interviewed. This study was approved and certified exempted by the East Carolina University Institutional Review Board.

### 2.2. Data Collection

Interviews took place between October 2020 and April 2021. Following the verbal informed consent process, each participant completed a brief demographic survey verbally. After completing the demographic survey, an open-ended interview was conducted. Interview guides were created by the first and second authors and were developed based on the study aims and authors’ previous qualitative work in the eNC region [13,14,25]. Sample questions from each interview guide follow: (1) Riders: Thinking back to a time before you used Project TRIP for transportation, how would you get somewhere you needed to go? Can you explain how Project TRIP works, the process to schedule a ride? What would you keep the same and what might you change about TRIP if you could? (2) Staff: Can you describe to us how Project TRIP works overall from beginning to end with a patient? In your perspective based on your role as a [insert role here], what would improve TRIP and make it a better program? What aspects would you retain and why? and (3) Transport providers/drivers: Can you explain to us how being a driver for Project TRIP works? For example, how do you know who and when to pick up? What do you like most about being a driver in Project TRIP? What do you like least about being a driver in Project TRIP? Is there information you wish you were told about Project TRIP prior to getting involved as a driver that could have better prepared you to serve the riders?

Interviews were conducted by the first three authors (AJS, ARR, and MS), lasted approximately 30–45 min each, and were digitally recorded. Following the interview, the participant was mailed a $25 gift card to a local grocery store as a thank you for their participation. Transcription was completed by MS. 

### 2.3. Data Analysis

A thematic analysis approach was used to analyze the data [26]. AJS read all transcripts and completed open coding identifying words or phrases of interest during the initial read [27]. In the succeeding read of the transcripts, the open codes were then organized by relationships into axial codes [27]. ARR read 5 randomly selected transcripts and met with AJS to compare coding. Any disagreements about coding between AJS and ARR were resolved by reaching a consensus via conversation. After all transcripts were coded by hand, AJS uploaded the word version of each document into NVivo 12 Software [28], where she coded the transcripts again in the software using the agreed upon codes. NVivo facilitated the sorting and identification of additional relationships between codes. This also led to the development of a coding framework facilitated by the software and continued the audit trail from paper to computer, providing further detail of the analysis process [26]. Axial codes were collapsed into what became the study themes [27]. The use of NVivo software enabled creating output for each theme by collating all quotes associated with each theme into a document. Using NVivo in addition to the initial hand coding process further enhanced familiarity with the data [26], and supported “working more methodically, more thoroughly, [and] more attentively” (Bazeley & Jackson, 2013, p. 3) [29]. To ensure agreement across these processes and the development of themes, AJS would review the themes and output during team meetings with the co-authors and ensure a consensus agreement was reached [26].

Throughout the data collection and analysis process the researchers practiced reflexivity- an intentional awareness and exploration of our biases, opinions, and world views. Doing so supported our openness to the data and what it might tell us, versus incorrectly interpreting data based on our own experiences [30,31]. The authors implemented elements of group reflexivity when meeting weekly to debrief and inform each other about the interviews conducted and any surprising, shocking, or even upsetting information discussed in the interviews. There were additional communications between team meetings via telephone and/or noting personal thoughts after completing interviews and reading transcripts [31]. 

During the analysis process, data saturation was met for the staff and rider interview data. No new ideas or concepts emerged from the interviews and there was repetition on what was being discussed [30]. However, this was not the case with the transportation providers and was likely due to only interviewing two individuals. Although the findings cannot be generalized due to the use of qualitative methods, as explained by Lincoln and Guba (1985) the thick descriptions provided in the succeeding sections can assist in determining transferability of the study findings to similar low-income communities in rural settings [26,32].

## 3. Results

Table 1 and Table 2 provide descriptive data of the participants. TRIP riders were an average of 60.8 years of age (range 46–82), 83.3% were African American, and 75% were female. The majority (83.3%) of the riders interviewed were 53 years of age and older. Half of the participants received disability, and 25% of riders were retired. Perceived income was measured, and 41.7% reported a “1”, indicating that they “were having difficulty paying the bills, no matter what they do”. Approximately 50% of the riders had less than, some, or a high school diploma or GED. 

Of the TRIP staff interviewed 83.3% were female and worked full time. Approximately 33% earned a college degree or a graduate degree (33.3%). The majority of staff were African American (83.3%). Of the two transportation providers interviewed, both were African American and were working full time. One person had a high school diploma or GED and the other had a college degree. Three overarching themes emerged from the staff, rider, and transportation provider interviews: (1) Emotional, health, & financial impacts of TRIP, (2) Changes that should be implemented into TRIP when replicating the program, and (3) Unique aspects of how TRIP operates that could inform other rural transportation programs.

### 3.1. Theme 1: Emotional, Health, & Financial Impacts of TRIP

*Financial Impact*. Most participants spoke about the positive financial impact TRIP had on their life or the life of their patients. A transportation provider who was part of the initial development of the program explained how “*The TRIP Program it’s very different from the other contracts that we have…the difference is we are transporting people that do not have a fund to be able to provide transportation for themselves…*” A patient explained how prior to TRIP they had to “*…beg for people to take you somewhere and if you ain’t got money, you couldn’t go…*” Depending on where the person was traveling to, costs could vary. One patient was told by someone they asked, “*Well if you don’t got $50, I can’t take you. I’ve gotta have gas to get there and back*”. Thus, in some instances people might miss appointments and doing so could be detrimental to their health. For example, TRIP enabled a patient to adhere to a rigorous cancer treatment schedule without being concerned about arranging or paying for rides,

*…a person may charge $30 or…$40… sometimes I didn’t have it…every week, I might be going out there 7 different times…since I’ve been back due to the cancer, I have been truly blessed that I haven’t missed not one appointment*.

Like patients, transportation companies reported financial benefits from their involvement in TRIP. Serving as a transportation provider helped local businesses grow, which is of economic relevance considering the areas that TRIP serves are Tier 1 counties. One transporter explained how TRIP “*…helped me to get my company established… the income and the pay… it also helped taught me um good people skills…to deal with different people…*” This participant also reported that TRIP’s pay per mile was competitive with Medicaid reimbursement rates. The contribution to economic development was noted by a TRIP staff interviewee. “*When you work with the drivers making sure to kind of document how it’s impacting them financially…and their business growth. Um, if they’re able to you know hire more drivers, or if there’s an increase in their revenue…[that’s] good*”.

*Health*. As stated above, prior to TRIP, patients who could not afford or did not have someone to transport them, would miss appointments that in turn affected their health and wellbeing. When asked to explain a time they could not get to the doctor and how they resolved that issue one patient stated, *“I couldn’t go…it won’t [sic] nothing I could do”*. A staff member reiterated this, explaining how TRIP provided direct access to health care, particularly for those who “*… hadn’t been to appointments in months or years because they didn’t have transportation… once the program started patients were going to appointments, they were getting resources they couldn’t get before… it was completely life changing*”. For example, as explained by a cancer patient, “*And if it won’t [sic] for it [TRIP], I may not be alive. It’s a part of saving my life… It provided me to get to radiation and chemo treatment*” A case manager emphasized how vital TRIP was. “*We transport to the Oncology department, the primary care … other appointments … such as dental appointments, eye doctor… their life depends on this…*”

*Emotional health Impacts*. In addition to physical health impacts, TRIP stakeholders reported positive impacts on patients’ emotional health. One patient reported a decrease in stress,

*… it’s less stress because I had to sit here and figure out how I’m gone [sic] get from point A to point B and back again in time for me to do something else that I need to take care of … been less stressful than it was when I was with social services … with that my blood pressure and all that has come down to a good level*.

A daughter who would accompany her mother on rides to appointments reported the impact on her mother’s emotional health,

*… her siblings would help…And they definitely would take time out, but you don’t want them to miss too much on their job … it’s definitely been a peace of mind for her and a comfort… she knows they’re coming and it’s gonna be a pleasant ride …*

One rider discussed transportation provided by social services prior to TRIP and how it was unreliable.

*There has been a time where the driver forgot to come and pick me up…and can’t get a hold of that driver … I noticed that in 30 min if my appointment is an hour away, I won’t gone make it, so I have call back to the doctor’s office and reschedule … but I now make it to all my doctor’s appointments with TRIP*.

Prior to the COVID-19 pandemic, TRIP would also take patients to places for socialization or overall well-being such as gyms.

*… I felt less isolated. Um, I was more able to socialize and maybe made me feel better to get exercise …It’s also less stressful to … be able to go somewhere… you really needed to go that you couldn’t … it’s kind of aggravating to have to worry about how you’re going to get somewhere*.

The experience of the ride itself positively impacted patients’ emotional health as well.

*…the driver played music, it’s very uplifting gospel… I got to be feeling down because of the conditions I’m in, and riding and listening to that music will lift me up… to get in that vehicle and be uplifted… when I get out of that vehicle, I’m thinking about that music…*

Due to drivers’ preparedness, patients could relax and enjoy the ride.

*… the doctor might be new, and I don’t know this doctor, but she [driver] does. She calms me down; she gets the address and the phone number…so its been great…we have a conversation on the way there … I learn a lot listening to them and they learn a lot from me, so it goes both ways*.

The drivers’ personalities were an integral part of a stressless and enjoyable ride, where the patient could enjoy the driver’s company. “*…it just came about so naturally like I been knowing them all my life…the way they care… the way they talk to you… they really care about your … health situation and what you’re going through*”. In one circumstance, the driver and the patient did know each other, but this only enhanced the experience for the patient. “*…She is very sweet and supportive …I didn’t know she was gonna be my driver… When she drove up it’s like I got a present for Christmas… we old classmates and we was friends from elementary school…*” Patience, respect, and caring were key adjectives used to describe drivers and their impact on patients. “*They take their time and be patient with you. And they really care… when you is at your worst and when you have someone that’s giving you a word of encouragement, it lifts you up*”. And stated by another patient, “*They take their time to take you…they give you a conversation and they’re nice talking to you… They always give me respect*”.

### 3.2. Theme 2: Changes to TRIP to Implement when Replicating the Program

Although mostly positive insights were shared about TRIP, all stakeholders suggested changes to improve the program. This was particularly important considering the first and second author’s plans to replicate and test TRIP in another eNC-29 county. In the interview process, case managers explained how TRIP was an added piece of responsibility on top of their current duties. One case manager noted that “*…the TRIP piece should be something separate… have their own coordinators for … scheduling trips…Making sure everybody is getting to where they need to go… it would be a job by itself*”. This was echoed by TRIP staff who indicated that more staffing and community engagement was important.

*… more staffing … we need to do a better job of reporting it out and getting it on the agenda. But I think having the community behind it more… the community hasn’t really grasped it like they should… for it to be such a good program I feel like … it needs to be more represented in the community*.

Through the stakeholder interviews the authors learned that transportation companies are paid monthly for their services, and this was noted as problematic by one transportation company. “*… thing that I liked the least was… getting the monthly revenue…When you got another driver that is working for you … you don’t want that driver to have to wait 30 days to get paid*”. In addition to the payment timeframe, other commentary addressed the individualized nature of the program. A transporter stated,

*…you take them to the doctor’s appointment…you wait for them…that one person…the only thing that I really didn’t like about that process was um when you have one person then you can’t take another person… Medicaid and Medicare ya know, you can carry more than one client at a time…*

Some of the rules included participants not being able to eat in the van during travel. Patients thought this warranted change based on individuals’ circumstances/health needs.

*…when we made those long-distance trips they go straight there, straight back…we can’t bring food …. now that I’m a diabetic I’m gonna need to eat… if we can just stop by and get a sandwich or something like that on our way back home that would be better*.

Despite this patient’s experience, per the patient responsibilities form that individuals sign at the time of enrollment, it is noted that patients may eat in the vehicle if they have a health condition that warrants this, such as diabetes. It is possible that the driver and/or the patient did not understand some of the travel allowances, and clarification may be needed.

Although patients praised many transportation drivers, there were some reports indicating challenging or stressful travel experiences due to drivers’ behaviors. This suggested that driver training would be warranted. One rider noted how, “*… some of the drivers were acting like they were doing me a favor…they were kind of condescending. Like [you] weren’t always out there when you got out… they expected you to be waiting for them…*” Another rider described a behavioral issue with a driver.

*… one of the male drivers tried to get um sexual with me… It really messed me up … they fired the company… we’ve been friends for a long time … talking and stuff… out of the blue…he decided to act up…it shocked me, it really put me in a position that I didn’t want to be in, and it’s very stressful*.

One of the staff interviewees referred to the same incident reported by the rider above and stressed how driver training was warranted.

*… when we open up to more companies having some training to the men about um you know um keeping… professional….there’s still a fine line that people just don’t get because you know you’re just being friendly making small talk, and then it be kind of going to something else…this one particular person…she had actually known the driver…And then he got inappropriate with her…so they’re bringing in all this outside stuff also that you’re not aware [of]*.

The same staff member continued to stress the importance of the drivers and training and how they are an integral part of the care team. Such changes to TRIP could include training to engage drivers further in the care process:

*… drivers can play a major part in their health care. So maybe like some… motivational interviewing type skills. That could possibly be able to help the case manager or help their provider…I want to work on that two-way communication because I feel like they are going to the home and they are seeing …they have a huge hole in their roof or something like that*.

Inclusion of the drivers in additional ways is discussed further in Theme 3 regarding TRIP operation details.

### 3.3. Theme 3: Unique Aspects of TRIP That Could Inform Other Rural Transportation Programs

Project TRIP is unique in its individualized rides with the driver waiting until the patient is done to take them directly home or to their next destination. Interviewees highlighted other details regarding TRIP program operations that made it particularly successful. This information is useful in developing other rural transportation programs to increase access to healthcare.

*Collaboration between drivers and case managers*. Collaboration between the drivers and case managers was readily apparent in the interviews, including drivers going above and beyond to ensure patient safety. A case manager described her working relationship with a driver and how it was imperative to the patient’s health,

*This one vendor she has really, you know we have very good communication…And because she gets to know these patients, when you’re driving them to multiple appointments…She’ll call me and say “…Something’s not right with this patient” … she knows when they don’t look well, or they don’t feel well…that’s not a part of her job description, but she’s a very good human being…*

Although not a part of their job description, the driver reported in how they felt it was important for them to be in the “middle”, and facilitate contact between the patient and case manager.

*… we reach over a little bit further with the patients because we understand that there’s a lot of barriers of just no income, some of them are uneducated…we do it because we care … we make sure that the case managers are aware of the status of their patients… to keep everything on point and keep it so that they can keep their appointments, so that their case manager can be aware of what’s going on with their patients. So, we’re like in the middle. And we make it a point to report to the case managers anything that we observe or notice about their patients …. I think we have a great working relationship. They listen and they understand…we want to work together …*

Another staff member noted the importance of the driver in identifying health related issues going on with patients but other issues of concern within the context of being situated in a rural community.

*…especially because these are such small communities, and people are born there and still live there…knowing what those professional boundaries and expectations are but also…assessment skills. Like if you pull up to this person’s home and there is a hole in the roof, please report it…note what isn’t okay and that it’s okay to share with the case manager… their role involves as a professional, it’s not just driving*.

The second driver interviewed for this study echoed some of the comments above regarding the relationship between drivers and case managers:

*I got a chance to meet the people involved with … handling TRIP. Then I got a chance to meet the patients…I started a relationship with the people that I uh transported… I started a relationship with the case manager who was in charge of the people that I carry*.

*One point of contact*. Navigating the healthcare system is problematic for many individuals and not having transportation further complicates the process. TRIP processes are streamlined with patients having one point of contact, their case manager. The process was described succinctly by both staff and transportation stakeholders.

*… the case manager will call us, and they will request transportation for a patient…we tell them to email us over the ticket… it will have the patient’s name, the patient’s ID, the patient’s address, the day of the appointment, the time of the appointment, and the name of the visit… We’ll let them know if we can take it based on our schedule and then after that, what we do is we set up the transport…the day of the transportation or the day prior to transportation … we’ll call the patient and we’ll let them know that … you have an appointment tomorrow at 10 o’clock am, you’re going to name of city and we will arrive at your place to pick you at such and such time…*

As described by a case manager, the process can be more complicated depending on the patient.

*Some patients are a little more … self-sufficient or responsible and they’ll call you and let you know. And then other times it’ll just be if I’m completing a status check and I ask about any appointments, future appointments then they’ll say something about it. But we do let them know to give us um their appointment in a timely manner…*

From the patient point of view, the process is simple. “Oh, it works out great. I call her [case manager] every time I get an appointment…so she can put it down on the calendar…” It takes one call and from there all follow up calls are provided by the case manager and/or transportation provider.

*…[you] call in and schedule for your ride they will let you know… who gonna take me … they’ll have that same person that gone [sic] take me to call me and let me know they gone pick me up*.

Another patient explained how having her case manager as the main point of contact made her feel supported. She stated, “…[Case manager] has no problem in keeping me in line. And I forget too because I have multiple sclerosis … she has my back”.

## 4. Discussion

As indicated in previous literature, if patients do not have access to funds for transportation, or access to transportation at all, they may opt to forgo or not schedule appointments, which been identified as a key factor among eNC rural residents in previous literature for missed appointments [12,13,14]. In some instances, this can be life-threatening given the severity of some diagnoses (e.g., cancer). Like other studies, the present research also found how one point of contact is crucial in streamlining and supporting individuals’ navigation of the often-fragmented healthcare system, that can result in positive health outcomes [33]. TRIP provides patients with transportation while streamlining the appointment scheduling process to ensure adherence to appointments and other healthcare related locations. Our research also highlighted the collaboration between case managers and drivers. Although they are not medical professionals, drivers in Project TRIP are part of the patient’s care team, and their relevance, knowledge, and skill were essential in meeting patient success. This was particularly important to the team as we began the expansion of Project TRIP in another eNC-29 county, Washington County in summer of 2022. Although we are aware drivers’ feedback and observations are important, the involvement of drivers in supporting patient success and health remains understudied.

Stakeholder narratives identified the stress patients experience in getting to appointments and contributed to understanding how the transportation itself alleviated stress and had positive impacts on health. The actual ride was an integral part of patients’ experiences and of equal importance in increasing patient access to healthcare. Patients reported developing a rapport with the drivers, decreased social isolation, and feeling “uplifted”. This may be a unique characteristic of TRIP, as one of the transportation companies was involved in the development of the program. In addition, the fact that the transportation company owners live in the community they serve may contribute to their understanding of local cultural norms and they may be particularly invested in residents’ health. As the transportation companies may know some patients due to rural towns being smaller, this may be a factor in building trust between the residents and transportation providers. Also of note was how TRIP has the added benefit of contributing to local economic development, and this is particularly poignant considering the Tier 1 counties where service is taking place.

The present study had limitations that present opportunities for future research. Given the qualitative approach used in the research, we cannot generalize the study findings to all settings that have transportation needs. However, with the thick description of the data that was provided from the stakeholder interviews, the transferability of the findings to similar rural low-income communities is possible. Only 2 interviews were completed with transportation companies. At the time the research was conducted there was only one transportation company serving TRIP. Although other companies previously serving TRIP were contacted, they declined to be interviewed. It would have been beneficial to have interview data from additional drivers. This also impeded the ability to reach data saturation from the transport provider perspective. Despite this, there is scant literature that includes drivers and considers the positive or negative role they play in riders’ experiences [34], and additional research including them is warranted. Other research should examine the role of peer training, or drivers training drivers. Training could implement assessment skills, motivational interviewing, interprofessional education, and tools such as simulation [35,36] and test the effectiveness of such training on the service drivers provide.

It is also important to highlight how the data was collected during the COVID-19 pandemic and the possibility of this having impacted participant responses. However, the authors do not think the pandemic influenced responses regarding participants’ likes or dislikes about their involvement in TRIP. However, an explicit examination of the impact of the pandemic on stakeholder opinions of TRIP or other transportation programs warrants exploration in future research.

Although successful, TRIP remains a smaller program serving specific counties in the eNC-29 subregion. Efforts to expand should be considered, including the integration of technology to further support the program. As described by one TRIP staff stakeholder, patients have access to MyChart, a website where they can communicate with their care team, access health data, etc. Integrating TRIP into the application so that patients could request visits and sign up for trips simultaneously, may support streamlining the program further, while simultaneously empowering patients to engage in their care. This would require research regarding the feasibility of this option, given the issues regarding limited internet access in rural communities.

As discussed earlier, most of the riders interviewed were over the age of 53 and the counties being served by TRIP are predominantly low-income with health disparities most evident among BIPOC individuals. Although we interviewed TRIP riders at one point in time, it is important to consider how their current challenges in accessing care and places for well-being may be attributed to cumulative disadvantage experienced over their life course [22] and within the context of living in a rural setting. Even though TRIP users are not solely “older adults”, TRIP is vital in intervening at various points in patients’ lives making it possible that the program may counterbalance some of the health-related disadvantages people experienced across the life course. It is possible that this could impact health in later life, however, a longitudinal approach is warranted to determine if TRIP has such an impact from adult through later life stages [37,38,39].

## 5. Conclusions

In sum, our evaluation of Project TRIP found that the transportation program has positive impacts on emotional, physical, and financial health of riders. Despite this, through stakeholder interviews we were able to identify areas of the program that could be modified for further improvement and aspects of TRIP that were unique—both which could be used to inform the replication of TRIP in other locations. This included having more staff and those solely dedicated to case management needs for the program, bimonthly pay to transportation providers, and clarifying the patient rules for participation in the program. Providing drivers with training was also noted as key given their role in the program, as discussed above. Aspects of TRIP that other rural transportation programs should consider during development stages included the promotion of collaboration between drivers and case managers and ensuring that case managers are the key point of contact for riders.

TRIP can inform the development of sustainable transportation solutions for rural residents, such as reimbursement through health insurance companies for travel given the positive outcomes (e.g., adherence to appointments). The stakeholder interview data is crucial in expanding and further testing TRIP to identify impacts to healthcare utilization, along with other methods in the overall mixed methods study. We have already capitalized on the knowledge gained from the stakeholder interviews and have applied what we have learned in an expansion pilot currently being tested in Washington County, NC. In conclusion, to date, our study has found that TRIP, a transportation program serving low-income residents as they age in rural eNC has supported access to healthcare and improved the physical and mental health of residents.

## Figures and Tables

**Table 1:** Characteristics of TRIP Riders (N = 12).

	Mean (SD), Range	%
Female		75.0
Age	60.8 (9.9), 46–82	
African American		83.3
White		16.7
Never married		41.7
Widowed, not currently married		25.0
Divorced or separated		33.3
Working part time		8.3
Not currently working		16.7
Retired		25.0
On disability		50.0
Less than high school		8.3
Some high school		8.3
High school diploma/GED		33.3
Some college		16.7
Associate degree		25.0
Graduate school		8.3
Perceived Income ^a^		
1		41.7
2		25.0
3		25.0
4		8.3

^a^ 1 = Having difficulty paying for bills, 2 = Have money to pay bills, but cut back, 3 = Have enough to pay bills, little to spare, 4 = After paying bills, have enough for extras wanted. Note. Percentages over or slightly under 100 are due to rounding.

**Table 2:** Characteristics of TRIP Staff & Transportation Companies (N = 8).

	Staff (n = 6)	Transport (n = 2)
	%	%
Female	83.3	50.0
African American	83.3	100.0
White	16.7	--
Working full time	83.3	100.0
Other working status	16.7	--
High school diploma/GED	--	50.0
Associate degree	16.7	--
College degree	33.3	50.0
Some graduate school	16.7	--
Graduate school	33.3	--

## Data Availability

The data are not publicly available due to sensitivity of human subject data and are available from the corresponding author upon reasonable request.
